# Ependymal cell inflammatory activation in response to intracerebral hemorrhage

**DOI:** 10.1186/s12974-026-03809-z

**Published:** 2026-04-14

**Authors:** Jing Liu, Haopu Lin, Xuefeng He, Yonghe Zheng, Jianan Wu, Qizhen He, Cheng Zhang, Huaping Huang, Xian Yu, Yinan Zhou, Zihang Chen, Hang Zhou, Linfeng Fan, Xiongjie Fu, Tianchi Tang, Guannan Guan, Xiaobo Yu, Xiuqin Feng, Yushen Du, Huaijun Chen, Jingyin Chen

**Affiliations:** 1 Department of Nursing, the Second Affiliated Hospital, School of Medicine, Zhejiang University, Jiefang Road 88th, Hangzhou, Zhejiang 310009 China; 2 Department of Neurosurgery, the Second Affiliated Hospital, School of Medicine, Zhejiang University, Jiefang Road 88th, Hangzhou, Zhejiang 310009 China; 3 Zhejiang Key Laboratory of Research and Transformation for Major Neurosurgical Diseases, Jiefang Road 88th, Hangzhou, Zhejiang 310009 China; 4 Cancer Institute (Key Laboratory of Cancer Prevention and Intervention, China National Ministry of Education), the Second Affiliated Hospital, School of Medicine, Zhejiang University, Jiefang Road 88th, Hangzhou, Zhejiang 310009 China

**Keywords:** Intracerebral hemorrhage, Ependymal cell, Complement pathway, Immune activation, Cilia-related function

## Abstract

**Background:**

Neuroinflammation is a central pathological process in secondary brain injury following intracerebral hemorrhage (ICH). While inflammatory responses in perihematomal brain tissue have been extensively investigated, the contribution of ependymal cells to post-ICH neuroinflammatory responses and ventricular pathology remains poorly defined.

**Methods:**

An autologous blood-induced ICH mouse model was used in combination with single-cell RNA sequencing and spatial transcriptomic analyses to characterize transcriptional reprogramming of ependymal cells. EGFP transgenic mice were used to trace the infiltration of peripheral immune cells. Clinical data was used to assess the association between C3 and hematoma volume. Complement pathway activation was evaluated through integrated single-cell transcriptomic analysis, spatial transcriptomic analysis, immunofluorescence staining, and western blot. *Bst2*-deficient mice were employed to investigate the mechanisms how complement pathway is regulated in ependymal cells.

**Results:**

Single-cell and spatial transcriptomic analysis showed impaired ciliary function and reduced capacity for homeostatic maintenance in ependymal cells. Ventricular asymmetry showed positive association with cognitive impairment at 14-days post-ICH. In parallel, ependymal cells underwent transcriptional reprogramming toward immune and inflammatory phenotypes, accompanied by ipsilateral immune cell infiltration. Cell-cell communication analysis further indicated extensive bidirectional signaling between ependymal cells and multiple immune cell populations, particularly through the complement pathway. Consistent with these findings, elevated C3 expression was detected in ipsilateral ependymal cells. Clinically, circulating C3 levels were elevated in patients with basal ganglia hemorrhage and positively correlated with hematoma volume. Knock out of *Bst2* downregulated C3 expression in ependymal cells.

**Conclusion:**

Following ICH, ependymal cells undergo a functional transition characterized by loss of cilia-dependent functions and gain of immune and inflammatory properties, including activation of the complement pathway, particularly C3, regulated by BST2.

**Supplementary Information:**

The online version contains supplementary material available at 10.1186/s12974-026-03809-z.

## Introduction

Intracerebral hemorrhage (ICH) is a severe subtype of stroke associated with high mortality and long-term neurological disability, for which effective disease-modifying therapies remain limited [1]. Beyond the immediate mechanical damage caused by hematoma expansion, accumulating evidence indicates that secondary injury processes, most prominently neuroinflammation, play a central role in determining neurological outcome after ICH [2, 3]. These inflammatory cascades involve rapid activation of resident glial cells, infiltration of peripheral immune populations, disruption of neurovascular integrity, and sustained alterations in brain homeostasis that can persist well beyond the acute phase [4, 5]. Recent studies have highlighted that ICH-induced inflammation is not confined to the perihematomal parenchyma but extends to ventricular structures, particularly in cases with ventricular involvement or proximity to the ventricular system [6]. However, the cellular and molecular mechanisms linking hemorrhage-induced inflammation to ventricular remodeling remain incompletely understood, limiting therapeutic strategies targeting these secondary consequences.

The ventricular interface represents a unique anatomical and functional niche within the central nervous system (CNS). Ependymal cells form a ciliated epithelial layer lining the ventricular system and play essential roles in cerebrospinal fluid circulation, metabolic exchange, and maintenance of ventricular homeostasis [7]. Disruption of ependymal integrity has been implicated in hydrocephalus, neurodevelopmental disorders, and neurodegenerative diseases [8–10]. Recent studies suggest that ependymal cells may also exhibit dynamic responses to brain injury [11], and their damage promotes neuroinflammation following intraventricular hemorrhage (IVH) [12]. However, the mechanisms by which ependymal cells contribute to neuroinflammatory signaling after ICH remain unknown.

Advances in single-cell and spatial transcriptomic technologies have now provided unprecedented opportunities to dissect cell type-specific inflammatory responses and intercellular communication networks in hemorrhagic brain injury. Although recent single-cell and spatial transcriptomic studies have begun to map the immune heterogeneity in ICH, ependymal cells are often underrepresented or primarily regarded as structural components [13, 14]. Single-cell transcriptomic analyses focusing on ependymal cells have mainly explored their stem cell-related functions, while their potential immunological roles remain largely unexplored [15].

Here, by integrating single-cell transcriptomic analysis with spatial transcriptomic validation, we identified multiple immune alterations involving ependymal cells after ICH, including chemotactic processes, immune activation, and engagement of the complement pathway. In addition, we observed impaired ciliary motility, disrupted intracerebral homeostasis, and consequent neurological dysfunction following ICH. Collectively, our findings indicate that ependymal cells are active contributors to post-hemorrhagic neuroinflammation and are associated with ventricular and neurological functional alterations.

## Methods

### Mice

Wild-type male mice (8-10 weeks) were purchased from Shanghai SLAC Laboratory Animal Co., Ltd (Shanghai, China). EGFP transgenic mice (Rosa26-CAG-EGFP; Cat. NO. NM-KI-190090) and *Bst2*-KO mice (Cat. NO. NM-KO-200058) were obtained from Shanghai Model Organisms Center, Inc (Shanghai, China). All mice were maintained under specific pathogen-free (SPF) conditions with free access to food and water. All animal procedures were approved by the Ethics Committee of the Second Affiliated Hospital, Zhejiang University School of Medicine.

### Autologous blood-induced ICH model

An autologous blood injection model of ICH was established as previously described [16]. Briefly, mice were anesthetized with isoflurane (3-4% for induction and 1.5-2.0% for maintenance). Autologous blood was freshly collected from the tail artery and immediately used for injection. After a midline scalp incision and skull exposure, a small burr hole was drilled over the target site. A total of 30 µL of autologous blood was stereotaxically injected into the right basal ganglia. The injection site was located 2.5 mm lateral to the bregma. The needle was advanced to a depth of 3.0 mm at an angle of 5° relative to the vertical axis, directed from lateral to ventral and perpendicular to the midline. The blood was infused at a constant low rate. After completion of the injection, the needle was left in place for 10 min before being slowly withdrawn. The burr hole was sealed, the incision was closed, and mice were allowed to recover on a heating pad. Sham-operated mice underwent the same procedure, including needle insertion, but without blood injection.

### Single-cell RNA sequencing (scRNA-seq) of brain tissue

Brain tissues were collected from mice at 3 days after ICH and from sham controls. The ipsilateral cerebral hemisphere was isolated for further analysis. To ensure consistency across samples and to focus specifically on the cerebrum, the olfactory bulb and cerebellum were surgically removed before tissue dissociation. Sham mice underwent the same anesthesia and stereotactic needle insertion procedures as the ICH group, but without blood injection. As reported in previous studies [17], 3 mice were included in each group (*n* = 3), and brain tissues from 3 mice in each group (sham and ICH) were pooled to generate scRNA-seq data. Single-cell suspensions were prepared using Neural Tissue Dissociation Kits (Cat. No. 130-094-802, Miltenyi Biotec, Germany) according to the manufacturer’s instructions, and cell viability was assessed prior to library construction. Single-cell RNA-seq libraries were generated using the 10x Genomics Chromium Next GEM Single Cell 3′ Kit v3.1 and sequenced on an Illumina NovaSeq™ X Plus platform, with sequencing services provided by Astrocyte Technology Co., Ltd. (Zhejiang, China). Raw sequencing data were processed using the 10x Genomics Cell Ranger pipeline to generate raw count data for downstream analysis.

### scRNA-seq data analysis

Raw data were processed using Seurat [18] in R. Low-quality cells were removed based on the number of detected genes and mitochondrial transcript content. After quality control, data from all samples were merged and normalized. Cell-cycle scores were calculated using a mouse cell-cycle gene set, and highly variable genes were identified. Data were scaled with regression of mitochondrial content and cell-cycle effects, followed by principal component analysis. Batch effects between experimental groups were corrected using Harmony [19], and the top principal components were used for dimensionality reduction and clustering. Cells were clustered using a shared nearest-neighbor graph and visualized by UMAP. Cell types were annotated based on canonical marker gene expression identified by differential expression analysis.

Differential gene expression between ICH and sham groups was performed using Seurat. Genes with an absolute log2 fold change > 0.25 and adjusted *P* < 0.05 were considered differentially expressed. Gene Ontology Biological Process (GO BP) enrichment was conducted separately for upregulated and downregulated genes using clusterProfiler.

Cell-cell communication analysis was performed using the R package CellChat (v1.6.1) [20]. Normalized gene expression data and cell-type annotations from Seurat analysis were used to infer intercellular communication networks based on the CellChat ligand–receptor database. Communication probabilities between cell groups were calculated using the default pipeline, and significant interactions between ependymal cells and other cell types were identified by permutation testing and visualized for downstream analysis.

### Spatial transcriptomics data analysis

Spatial transcriptomics data were obtained from a previously published dataset [14], which included both the autologous blood injection model and the collagenase-induced model of intracerebral hemorrhage. For the present study, only data from the autologous blood injection model were extracted for subsequent analyses. Samples in this external dataset were collected at 6 h, 1 day, and 7 days after ICH. And data were analyzed using Scanpy [21] and Squidpy [22]. To assess immune cell proximity to ependymal cells, spatial neighbors were identified using Squidpy with a radius of 25 μm. The average number of neighboring immune cells surrounding each ependymal cell was calculated for the ipsilateral and contralateral sides at different time points and visualized using violin plots. Spatial gene expression was visualized using color gradients based on expression levels. Cell type and regional annotations were kept consistent with those in the original dataset. Specifically, according to the original annotation, “infiltrated immunocyte” included B cells, dendritic cells, monocytes/macrophages, and neutrophils.

### Immunofluorescence staining

Mice were transcardially perfused with PBS followed by 4% paraformaldehyde (PFA). Brains were removed, post-fixed in 4% PFA overnight, and dehydrated in 30% sucrose at 4 °C [23]. The brains were then sectioned at 25 μm. Sections were blocked with 5% donkey serum (Cat. No. G1217-5ML, Servicebio, Hubei, China), and incubated with primary antibodies against C3 (Cat. No. sc-58926; 1:50; Santa Cruz Biotechnology, Texas, USA), FOXJ1 (Cat. No. 14-9965-80, 1:200; Thermo Fisher Scientific, USA), BST2/Tetherin (clone EPR23597-266, Cat.No. ab272169, 1:100; Abcam, Cambridge, UK), Iba1 (Cat. No. ab5076, 1:500; Abcam, Cambridge, UK), MAP2 (Cat. No. 17490-1-AP, 1:500; Proteintech, China) and cathepsin B (CTSB) (Cat. No. ab214428, 1:250; Abcam, Cambridge, UK), followed by Alexa Fluor 488/594/647-conjugated donkey anti-rabbit, anti-mouse, or anti-rat IgG secondary antibodies (1:1000; Invitrogen, USA), as appropriate. The sections were then mounted with a DAPI-containing mounting medium (Cat. No. ab104109, Abcam, Cambridge, UK). Fluorescent images were acquired using Leica confocal (Stellaris 5) and upright microscope (DM6B). Image processing and quantitative analyses were performed using Fiji/ImageJ (version 1.54p), and three-dimensional reconstruction was conducted using Imaris software (version 10.2).

Ventricular area was quantified using Fiji/ImageJ software (version 1.54p). In ICH mice, ventricular asymmetry was defined as the area difference between the ipsilateral and contralateral lateral ventricles.

For quantification of mean fluorescence intensity (MFI), images were analyzed using Fiji/ImageJ software (version 1.54p). The ependymal ROI was delineated on the basis of FOXJ1-positive ependymal cells, and MFI was quantified within the defined ROI. Detailed information on the time points included in each comparison, as well as the specific antibody used for MFI quantification in each experiment, is provided in the corresponding figure legends.

### Bone marrow transplantation

Bone marrow transplantation was performed to generate hematopoietic chimeric mice as previously described [24, 25]. EGFP donor mice served as bone marrow donors, and wild type (WT) mice were used as recipients. Recipient mice were preconditioned with busulfan (Cat. No. HY-B0245, MedChemExpress, New Jersey, USA) at 20 mg/kg by intraperitoneal injection on days -7, -5, and -3 before transplantation, resulting in a cumulative dose of 60 mg/kg. On day 0, bone marrow cells were harvested from the femurs and tibias of EGFP mice and transplanted into WT recipients via intravenous injection. Chimeric mice were allowed to recover and reconstitute hematopoiesis for 1 month.

### Agarose gel electrophoresis

For genotyping, genomic DNA was extracted from mouse tail biopsies. Briefly, approximately 2 mm of mouse tail tissue was collected and genomic DNA was isolated using the EasyPure Genomic DNA Kit (Cat.No. AD501, TransGen Biotech, Beijing, China) according to the manufacturer’s protocol. The purified DNA was used as template for subsequent PCR amplification. PCR products were analyzed by agarose gel electrophoresis (Additional file 5A). Briefly, amplicons were mixed with loading dye and separated on a 1.5% agarose gel prepared in 1× TAE buffer. After electrophoresis, DNA bands were visualized using a gel imaging system.

### Western blot analysis

Protein extraction was performed on brain tissue collected on day 3 after ICH. Tissue surrounding the hematoma was lysed in ice-cold radioimmunoprecipitation assay (RIPA) buffer supplemented with phosphatase and protease inhibitors (Cat.No.A32961, Invitrogen, USA). Protein concentrations were determined, and equal amounts of total protein were subjected to sodium dodecyl sulfate-polyacrylamide gel electrophoresis (SDS-PAGE), followed by electrophoretic transfer onto polyvinylidene difluoride (PVDF) membranes. Membranes were blocked with 5% non-fat milk in Tris-buffered saline containing 0.1% Tween-20 (TBST) for 1 h at room temperature and incubated overnight at 4 °C with primary antibodies against C1q (1:500, Cat.No. ab71940, Abcam, UK), C3 (1:2000, Cat.No. ab200999, Abcam, UK), and GAPDH (1:10,000, Cat.No. 60004-1-Ig, Proteintech, Hubei, China), followed by the appropriate HRP-conjugated anti-goat or anti-mouse secondary antibodies (1:10000, Proteintech, China). Protein bands were detected using enhanced chemiluminescence and quantified using ImageJ.

### Enzyme-linked immunosorbent assay (ELISA)

Levels of the membrane attack complex (MAC, C5b-9) in brain tissue lysates were quantified using a Mouse C5b-9 Terminal Complement Complex ELISA Kit (D721142, Sangong Biotech, Shanghai, China) according to the manufacturer’s instructions. Briefly, brain tissue lysates were prepared, added to pre-coated plates, and the assay was performed following the kit protocol. Absorbance was measured using a microplate reader, and MAC concentrations were calculated based on standard curves.

### Behavioral assessment

Cognitive function was evaluated using the Y-maze test at 14 days after ICH [26, 27]. The test was conducted in two phases. During the training phase, one arm was blocked, and mice were allowed to freely explore the other two arms for 5 min. After a 1-h inter-trial interval, mice were returned to the maze with all three arms open for a 5-min test phase. Exploration time and entries into each arm were recorded using an automated video-tracking system. Preference for the novel arm was quantified as the proportion of time spent in the novel arm relative to total exploration time.

### Clinical data collection

This study included 64 patients with ICH admitted to the Second Affiliated Hospital of Zhejiang University School of Medicine. The baseline clinical characteristics of the patients were presented in Additional file 1. ICH was confirmed by computed tomography, and all patients were admitted within 24 h of ICH onset. Peripheral blood C3 levels were obtained from routine clinical laboratory test results, and hematoma volume was assessed based on CT imaging. Patients with traumatic ICH, subarachnoid hemorrhage, infection, systemic inflammatory disease, malignancy, or severe cardiopulmonary, hepatic, or renal dysfunction were excluded. Clinical data were obtained from electronic medical records for subsequent analysis. The study was approved by the Ethics Committee of the Second Affiliated Hospital of Zhejiang University (ethics number: I2019001510).

### Statistical analysis

Normality and homogeneity of variance were assessed prior to statistical testing. For comparisons between two groups, an unpaired Student’s t-test was used when data satisfied assumptions of normality and equal variance. Comparisons among three or more groups were performed using one-way analysis of variance (ANOVA) when assumptions were met, followed by Tukey’s multiple comparisons test. Pearson correlation analysis was used to assess associations between variables.

## Results

### Overview of single-cell transcriptomic changes in the ICH brain

**Fig. 1:**
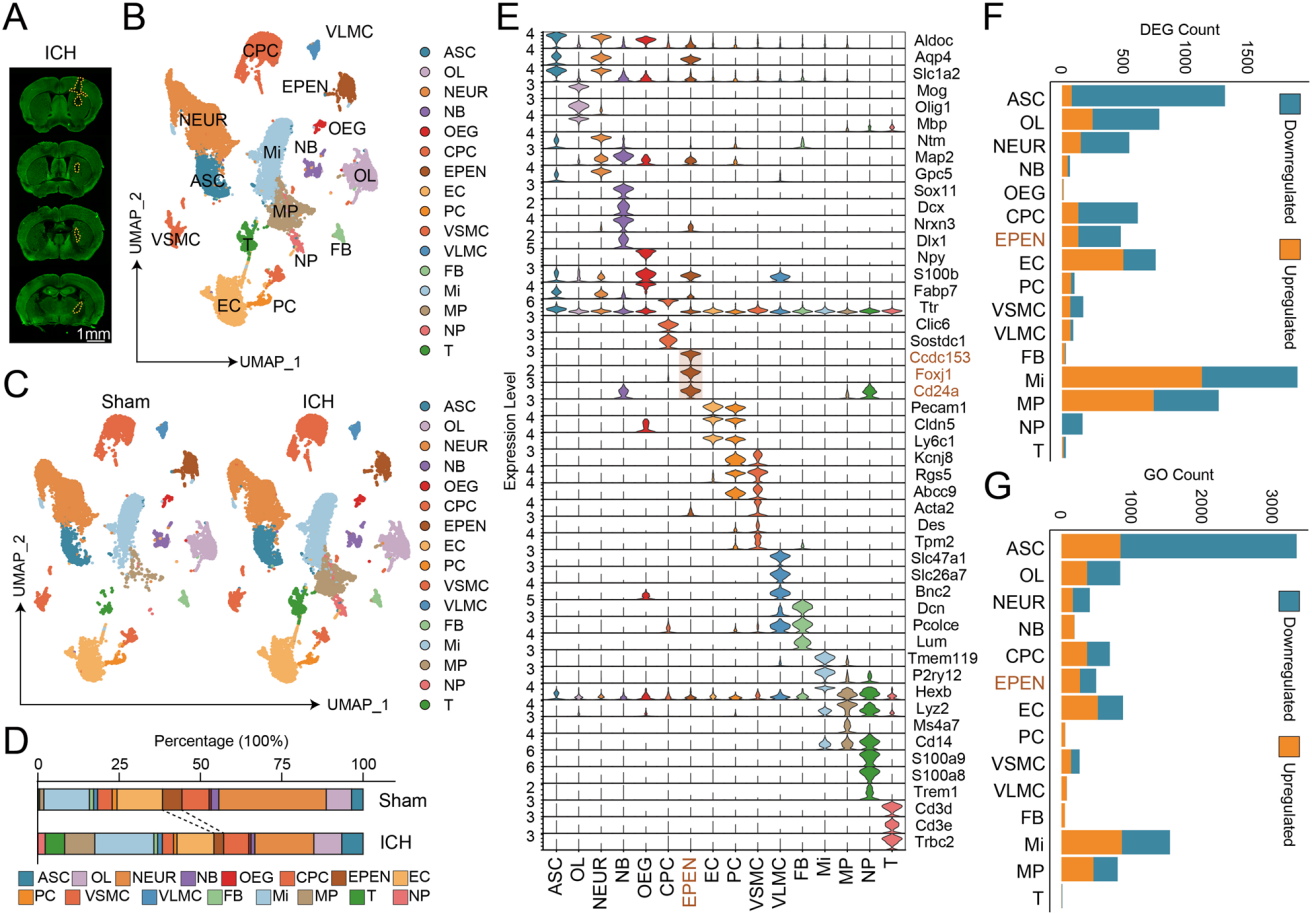
Single-cell transcriptomic analysis of sham and ICH brain tissue. **A** Representative MAP2 immunofluorescence staining of coronal brain sections at day 3 after ICH. Dashed circles indicate the peri-hematomal region. Scale bar, 1 mm. **B** UMAP visualization of all cells colored by annotated cell types, including astrocytes (ASC), oligodendrocyte (OL), neurons (NEUR), neuroblast (NB), olfactory ensheathing glial cells (OEG), chronic plexus cells (CPC), ependymal cells (EPEN), endothelial cells (EC), pericytes (PC), vascular smooth muscle cells (VSMC), vascular and leptomeningeal cells (VLMC), fibroblasts (FB), microglia (Mi), monocyte/macrophages (MP), neutrophils (NP), and T cells (T). **C** UMAP plots showing cellular distributions in sham and ICH (3 days) groups. **D** Stacked bar plots depicting the relative proportions of major cell types in sham and ICH brains. **E** Violin plots showing the expression of canonical marker genes used for cell-type annotation across all identified clusters. **F** Bar plots showing the number of upregulated and downregulated differentially expressed genes (DEGs) in each cell type following ICH. **G** Bar plots summarizing the number of significantly enriched Gene Ontology Biological Process (GO BP) terms for each cell type

To characterize cellular heterogeneity and transcriptional alterations following ICH, we performed scRNA-seq of brain tissues collected at 3 days after ICH and from sham controls. After quality control and integration, cells were visualized by UMAP and clustered into distinct populations representing major neural, glial, vascular, and immune cell types (Fig. 1A-C). These included astrocytes (ASC), chronic plexus cells (CPC), endothelial cells (EC), ependymal cells (EPEN), fibroblasts (FB), microglia (Mi), monocyte/macrophages (MP), neuroblast (NB), neurons (NEUR), neutrophils (NP), olfactory ensheathing glial cells (OEG), oligodendrocyte (OL), pericytes (PC), T cells (T), vascular and leptomeningeal cells (VLMC), and vascular smooth muscle cells (VSMC). Cell identities were validated by the expression of canonical marker genes across all clusters, including *Aldoc/Aqp4/Slc1a2* for ASC, *Mog/Olig1/Mbp* for OL, *Ntm/Map2/Gpc5* for NEUR, *Sox11/Dcx/Nrxn3/Dlx1* for NB, *Npy/S100b/Fabp7* for OEG, *Ttr/Clic6/Sostdc1* for CPC, *Ccdc153/Foxj1/Cd24a* for EPEN, *Pecam1/Cldn5/Ly6c1* for EC, *Kcnj8/Rgs5/Abcc9* for PC, *Acta2/Des/Tpm2* for VSMC, *Slc47a1/Slc26a7/Bnc2* for VLMC, *Dcn/Pcolce/Lum* for FB, *Tmem119/P2ry12/Hexb* for Mi, *Lyz2/Ms4a7/Cd14a* for MP, *S100a9/S100a8/Trem1* for NP, and *Cd3d/CCd3e/Trbc2* for T cells (Fig. 1E). Quantitative analysis of cell-type composition demonstrated a significant remodeling of the cellular landscape following ICH (Fig. 1D). Notably, the proportion of EPEN was markedly reduced in the ICH brain compared with sham controls.

To further define transcriptional responses at the cell-type level, we performed differential gene expression analysis and GO BP enrichment analysis between ICH and sham conditions for each annotated population. The number of upregulated and downregulated genes and pathways varied substantially across cell types, with immune and glial populations showing the most pronounced transcriptional changes (Fig. 1F-G).

### Ependymal cells acquire immune and inflammatory features after ICH

**Fig. 2:**
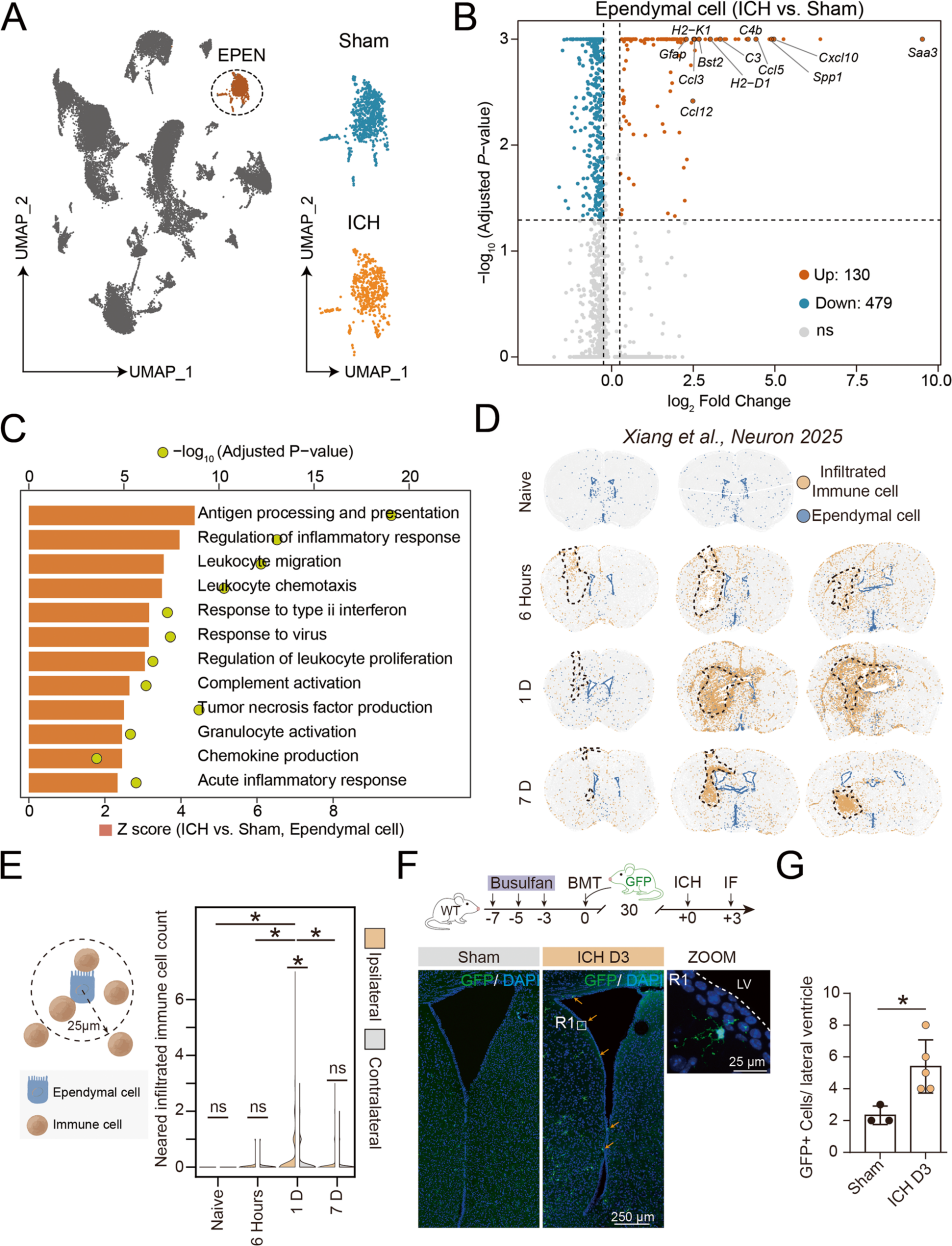
Immune activation of ependymal cells following ICH. **A** UMAP visualization highlighting the ependymal cell (EPEN) cluster and its distribution in sham and ICH brains. **B** Volcano plot showing differentially expressed genes in EPEN between ICH and sham conditions. **C** Gene Ontology (GO) enrichment analysis of DEGs in ependymal cells from the ICH group compared with the Sham group. Bar length indicates the Z score, and dot color indicates the -log_10_ adjusted *P* value. **D** Spatial transcriptomic analysis showing the distribution of infiltrated immune cells and ependymal cells at the indicated time points after ICH. Dashed outlines denote the hematoma region. Data were adapted from Xiang et al., *Neuron*, 2025 [14]. **E** Schematic illustration of the peri-ependymal region used for quantification and violin plot showing the number of infiltrated immune cells adjacent to the ependymal cells in the ipsilateral and contralateral hemispheres at Naive, 6 h, 1 d, and 7 d after ICH. **F**, **G** Schematic illustration of bone marrow transplantation and ICH induction and representative immunofluorescence images showing GFP⁺ peripheral immune cells in sham and ICH (3 days) groups. LV, lateral ventricle. Quantification of GFP⁺ cells in the peri-ependymal region (sham, *n* = 3; ICH, *n* = 5). Statistical comparisons between groups were performed using Student’s t test. *P < 0.05

Given the reduction of ependymal cells after ICH, we next investigated the transcriptional alterations of ependymal cells following ICH (Fig. 2A). Differential expression analysis identified extensive gene expression changes in ependymal cells following ICH, including upregulation of genes associated with immune activation and inflammation, such as *C3*, *C4b*, *Ccl3*, *Ccl5*, *Cxcl10*, *Ccl12*, and *Spp1* (Fig. 2B). Pathway enrichment analysis further demonstrated that ICH-induced transcriptional changes in ependymal cells were strongly associated with immune and inflammatory pathways, including antigen processing and presentation, inflammatory response, cytokine regulation, and granulocyte chemotaxis (Fig. 2C), indicating that ependymal cells acquire immune-related transcriptional programs after injury. To explore whether these transcriptional changes were associated with immune cell accumulation in vivo, we analyzed a publicly available spatial transcriptomics dataset [14]. Spatial mapping revealed a pronounced enrichment of immune cell including microglia and infiltrated immunocytes in regions adjacent to the ventricular-ependymal compartment following brain injury (Fig. 2D-E, Additional file 5B-C), with the infiltrating population being predominantly composed of myeloid cells, such as monocytes/macrophages (Additional file 5D). To validate immune cell distribution in the peri-ependymal region after ICH, we first performed FOXJ1/IBA1 immunofluorescence staining and observed increased IBA1^+^ cells infiltration after ICH (Additional file 5E). To further define the changes in peripheral immune cells, we generated hematopoietic chimeric mice by transplanting bone marrow from EGFP donor mice into WT recipients. Immunofluorescence analysis showed that GFP⁺ peripheral immune cells were already resident in the brain before ICH, whereas their contact with the lateral ventricle was significantly increased after ICH (Fig. 2F-G).

### Ependymal cells exhibit disease-associated microglia-like transcriptional features after ICH

**Fig. 3:**
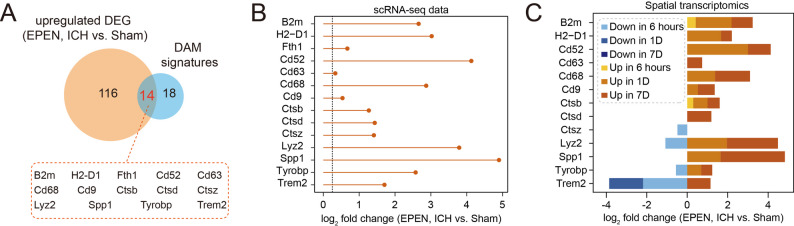
Disease-associated microglia-like signatures in ependymal cells following ICH. **A** Venn diagram showing the overlap between genes upregulated in ependymal cells after ICH and a curated set of disease-associated microglia (DAM) marker genes. **B** The log2 fold changes of overlapping DAM-associated genes in ependymal cells based on single-cell RNA-seq analysis. **C** The log2 fold changes of overlapping DAM-associated genes in ependymal cells at multiple time points, based on spatial transcriptomic analysis from a published dataset

Given the prominent immune and inflammatory transcriptional changes observed in ependymal cells after ICH, a substantial overlap was observed between ependymal cell-upregulated genes and disease-associated microglia (DAM) signatures [28] (Fig. 3A). The fold changes of overlapping DAM-associated genes at the single-cell level indicated acquisition of microglia-like disease-associated transcriptional features (Fig. 3B). To assess the spatial distribution of these DAM-associated genes, we examined their expression patterns using spatial transcriptomics data. Spatial mapping accordingly revealed that DAM-associated genes were expressed in the ependymal layer after injury (Fig. 3C, Additional file 2A-B). Among these DAM-related genes, we observed that CTSB, encoded by *Ctsb*, was upregulated after ICH (Additional file 2C). These results supported the notion that ependymal cells acquire disease-associated immune features in the injured brain.

### Loss of cilia-related functions in ependymal cells after ICH

**Fig. 4:**
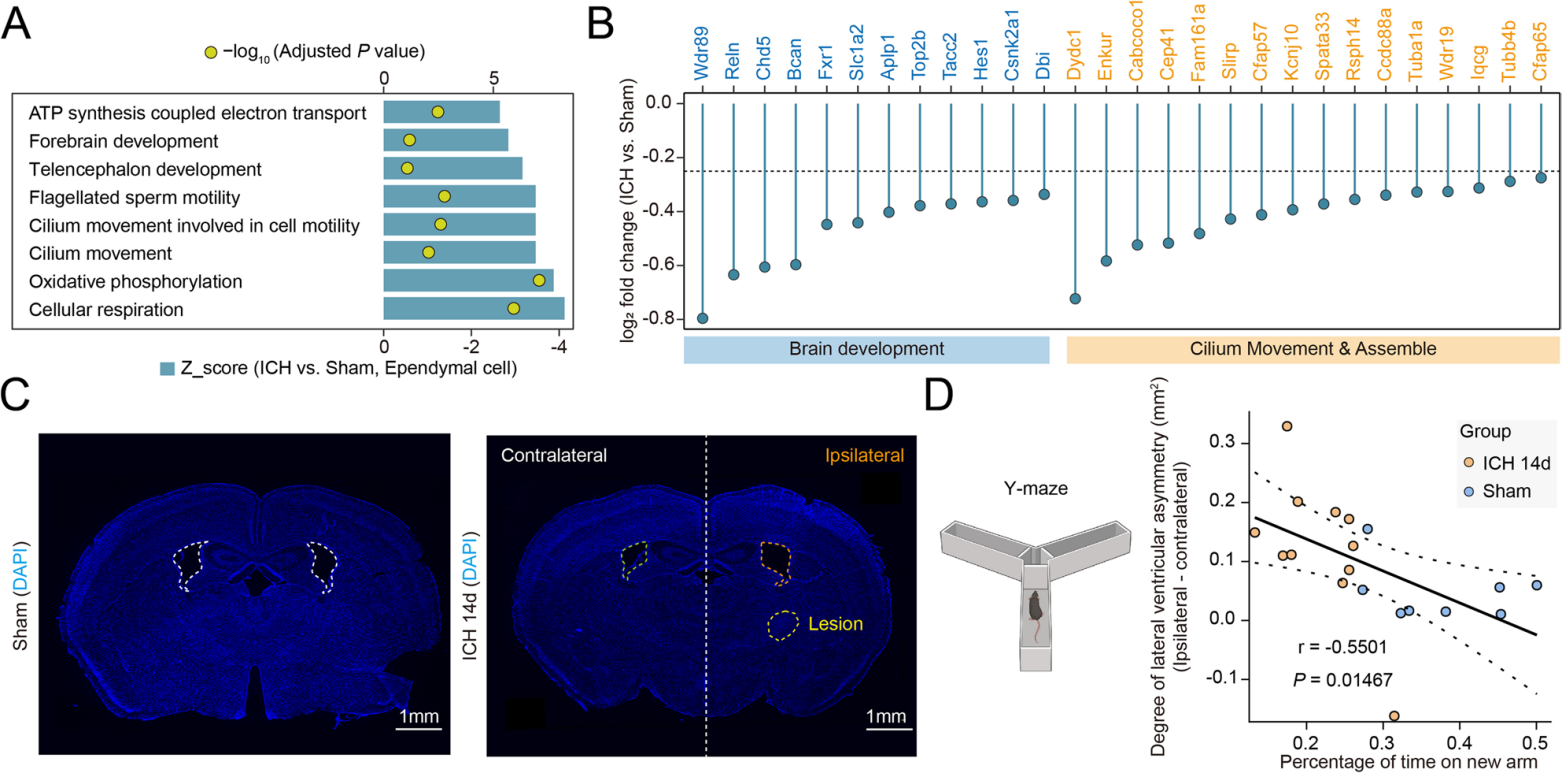
Impaired ependymal function following ICH. **A** GO BP analysis of genes downregulated in ependymal cells after ICH, Bar length indicates the Z score, and dot color indicates the -log_10_ adjusted *P* value. **B** Fold changes of representative downregulated genes in ependymal cells, particularly those involved in ciliary movement, cilia assembly, and brain development. **C** Representative DAPI-stained coronal brain sections from sham and ICH (14 days) groups. Dashed outlines indicate the lateral ventricle and the lesion area. Scale bars, 1 mm. **D** Left, schematic illustration of the Y-maze test; Right, correlation analysis of the association between the degree of ventricular asymmetry and Y-maze performance. Each dot represents one mouse. Sham, *n* = 8; ICH 14 d, *n* = 11. Correlation was analyzed by Pearson’s correlation analysis

We further analyzed the downregulated gene programs in ependymal cells after ICH. GO BP analysis of downregulated genes revealed significant enrichment of pathways associated with motility, neurotransmitter transport and uptake, brain development, indicating impaired ependymal structural and functional integrity after injury (Fig. 4A). Consistently, differential expression analysis demonstrated marked downregulation of genes involved in ciliary movement and assembly, and brain development (Fig. 4B). These transcriptional changes suggest a disruption of normal ependymal cell function in maintaining ventricular homeostasis after hemorrhagic injury.

Previous studies have shown that neurodegenerative diseases, such as Alzheimer’s disease, are closely associated with alterations in ependymal cell function and morphology [29, 30]. Accordingly, we assessed ventricular morphology and cognitive performance at 14 days after ICH to evaluate the potential anatomical and functional consequences of ependymal cell dysfunction [10]. Quantitative analysis revealed significant ventricular asymmetry in ICH mice compared to sham (Fig. 4C, Additional file 5G), suggesting ventricular enlargement on the ipsilesional side. Importantly, the Y-maze test indicated a marked decline in cognitive function in ICH mice (Additional file 5H). Moreover, the extent of ventricular asymmetry showed a strong correlation with Y-maze performance deficits (Fig. 4D).

### Enhanced ependymal cell-mediated chemotactic and complement signaling after ICH

**Fig. 5:**
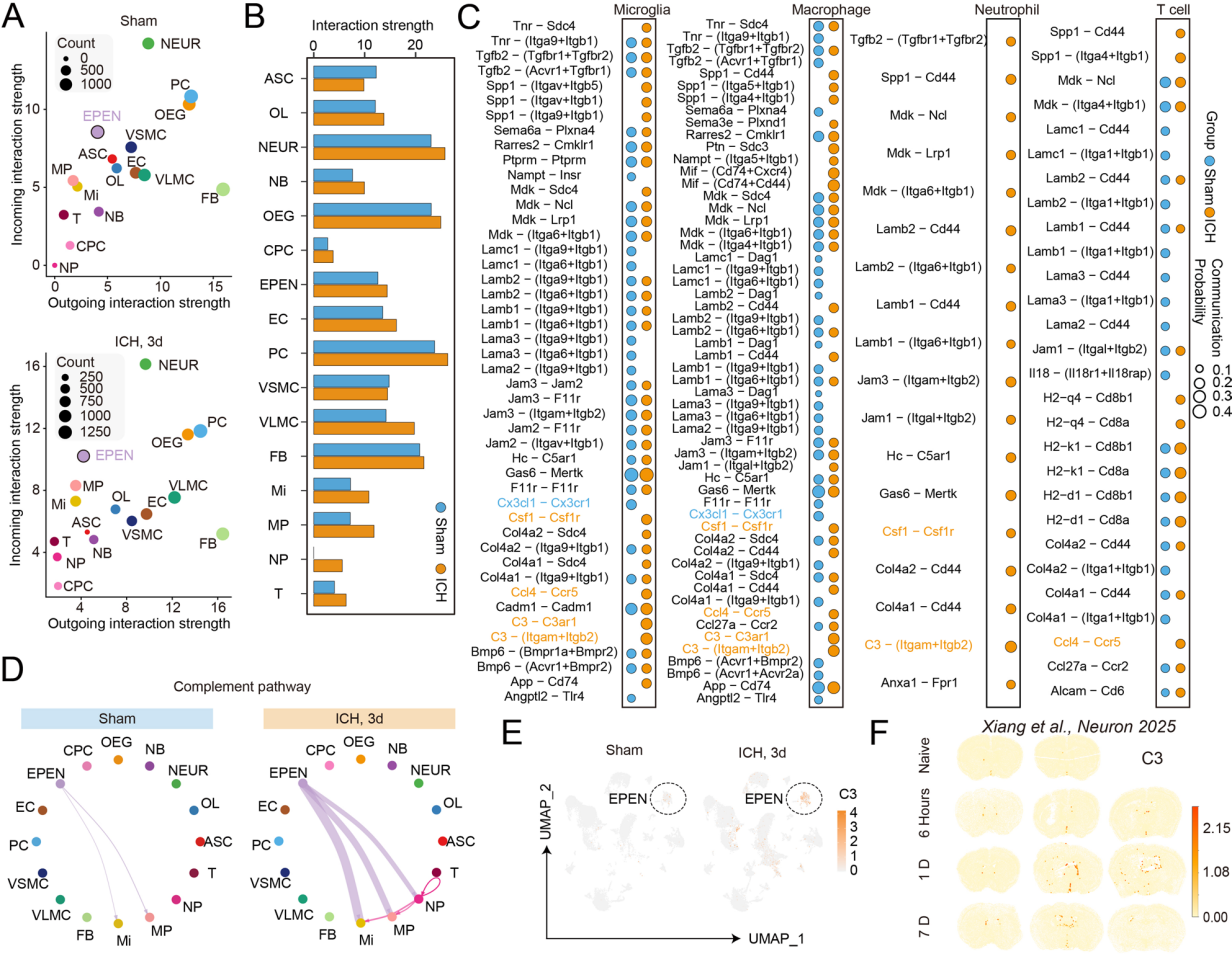
Cell-cell communication analysis identifies ependymal cells as a source of chemotactic and complement signaling after ICH. **A** Scatter plots showing outgoing and incoming interaction strengths of brain cell types under sham and ICH (3 days) conditions, including astrocytes (ASC), oligodendrocyte (OL), neurons (NEUR), neuroblast (NB), olfactory ensheathing glial cells (OEG), chronic plexus cells (CPC), ependymal cells (EPEN), endothelial cells (EC), pericytes (PC), vascular smooth muscle cells (VSMC), vascular and leptomeningeal cells (VLMC), fibroblasts (FB), microglia (Mi), monocyte/macrophages (MP), neutrophils (NP), and T cells (T). Dot size represents the number of inferred interactions. **B** Bar plots showing the overall interaction strength of the indicated cell types in the Sham and ICH day 3 groups. Bar color indicates group identity. **C** Dot plots illustrating significantly enriched ligand-receptor interactions between ependymal cells and immune cell populations. Dot size indicates communication probability, and dot color indicates group identity. **D** Network visualization of complement signaling pathways in sham and ICH (3 days) groups, highlighting ependymal cells as a source of complement-associated signaling after ICH. **E** UMAP feature plots showing increased *C3* expression in ependymal cells following ICH compared with sham controls. Dashed circles indicate the ependymal cell cluster. **F** Spatial transcriptomic visualization showing *C3* expression in ependymal cells following ICH in multiple time points. Data were adapted from Xiang et al., *Neuron*, 2025 [14]

To further investigate how ependymal cells may influence the post-ICH immune microenvironment, we performed cell-cell communication analysis to infer ligand-receptor interactions among major brain cell types. Compared with sham conditions, the overall communication landscape at 3 days after ICH exhibited increased interaction strength across multiple cell types, including ependymal cells (Fig. 5A-B). Pathway-level analysis revealed that ependymal cells preferentially engaged in signaling pathways related to chemotaxis, immune activation, and complement signaling, interacting with multiple immune cell populations including microglia, macrophages, neutrophils, and T cells (Fig. 5C). Notably, several complement-related ligand-receptor pairs, particularly those involving C3, were among the prominently enriched interactions between ependymal cells and immune cells after ICH. Consistent with these findings, complement pathway analysis demonstrated enriched complement-related signaling interactions between ependymal cells and infiltrating or resident immune cells following injury (Fig. 5D). Visualization of gene expression at the single-cell level further confirmed a marked increase in *C3* expression within the ependymal cell population after ICH compared with sham controls (Fig. 5E). Spatial transcriptomic analysis also revealed increased *C3* expression in periventricular ependymal cells after ICH, further confirming the above findings (Fig. 5F).

### Ependymal cell subclusters exhibit functional heterogeneity but display a shared inflammatory response after ICH

**Fig. 6:**
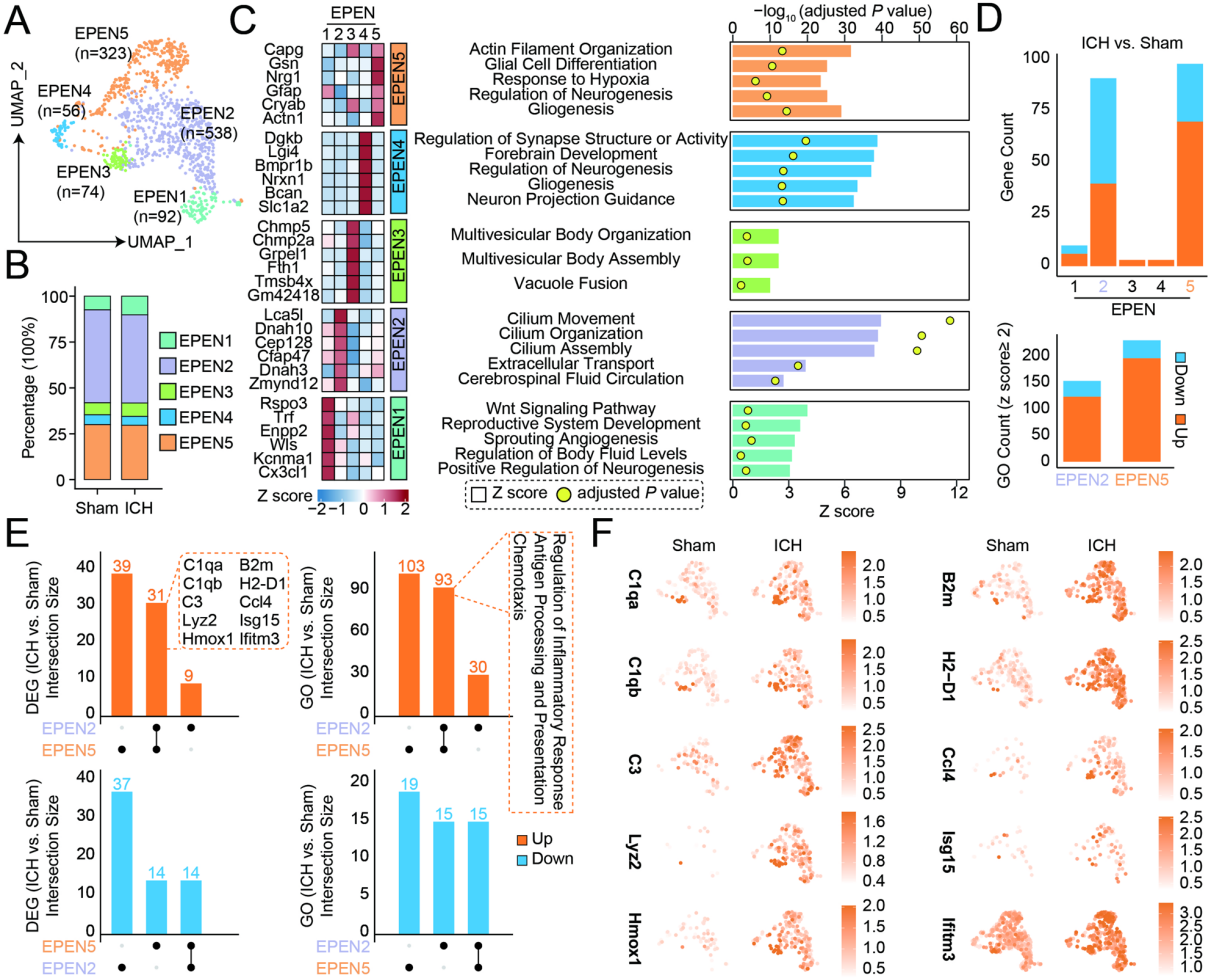
Subcluster analysis reveals immune activation across ependymal cell subpopulations after ICH. **A** UMAP visualization of ependymal cell subclusters (EPEN1-EPEN5). **B** Relative proportions of subclusters in sham and ICH brains. **C** Heatmap showing representative marker genes for each ependymal cell subcluster and Gene Ontology (GO) enrichment analysis showing the distinct functional programs associated with individual subclusters. Bar length indicates the Z score, and dot color indicates the -log_10_ adjusted *P* value. **D** Bar plots summarizing the numbers of upregulated and downregulated genes and GO terms in each subcluster following ICH. **E** Intersection analysis of differentially expressed genes and enriched GO terms between subclusters. **F** Feature plots showing increased expression of representative immune-related genes after ICH

To further resolve the functional heterogeneity of ependymal cells, we performed subclustering analysis of the ependymal population, identifying five transcriptionally distinct subclusters (EPEN1-EPEN5) (Fig. 6A). Quantitative analysis showed that the relative proportions of EPEN subclusters were largely preserved following ICH (Fig. 6B). These subclusters displayed distinct functional signatures, including enrichment of pathways related to ciliary movement and assembly (EPEN2), neurogenesis and brain development (EPEN1, EPEN4 and EPEN5), vesicle organization (EPEN3) (Fig. 6C). Instead, differential expression analysis revealed that transcriptional changes were predominantly driven by EPEN2 and EPEN5, which exhibited the largest numbers of differentially expressed genes after ICH, whereas other subclusters showed comparatively limited transcriptional alterations without significant pathway enrichment (Fig. 6D).

Notably, intersection analysis of differentially expressed genes and enriched GO terms revealed a substantial overlap between EPEN2 and EPEN5 (Fig. 6E). Shared upregulated genes and pathways were predominantly associated with chemotaxis, inflammatory response, antigen processing and presentation, and complement-related processes. Consistent with these findings, single-cell expression analysis showed robust induction of immune-associated genes, including *C1qa*, *C1qb*, *C3*, *Lyz2*, *Hmox1*, *B2m*, *H2-D1*, *Ccl4*, *Isg15*, and *Ifitm3* after ICH (Fig. 6F). Together, these results indicate that despite baseline functional heterogeneity, ependymal cell subpopulations develop a shared immune and inflammatory transcriptional response following hemorrhagic brain injury.

### Ependymal cell involvement in complement pathway activation after ICH

**Fig. 7:**
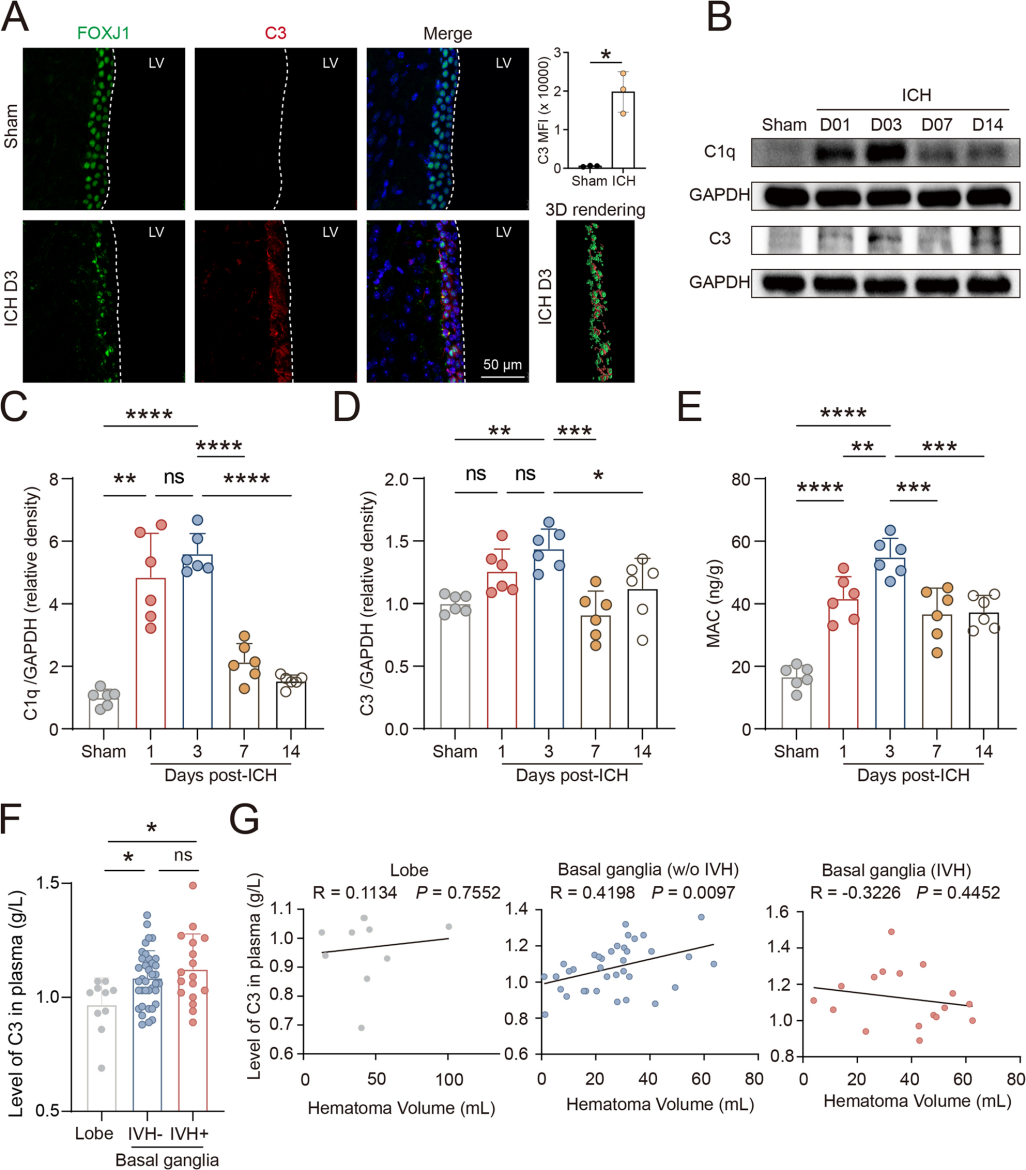
Activation of the complement pathway after ICH in experimental models and patients. **A** Representative immunofluorescence images showing FOXJ1 (green) and C3 (red) expression in sham and ICH (3 days) group, with corresponding merged images and 3D rendering. Quantification of C3 mean fluorescence intensity (MFI) in the ependymal-adjacent region is shown on the top right (*n* = 3 per group). LV, lateral ventricle. Statistical analysis was performed using Student’s t test. Scale bar, 50 μm. **B**-**D** Western blot analysis of C3 and C1q protein levels in mouse brain tissues from sham and ICH groups, and quantification of relative density of C1q/GAPDH and C3/GAPDH (*n* = 6 per group). Statistical analysis was performed using one-way ANOVA followed by Tukey’s multiple comparisons test. **E** ELISA-based measurement of membrane attack complex (MAC) levels in mouse brain tissues (*n* = 6 per group). Statistical analysis was performed using one-way ANOVA followed by Tukey’s multiple comparisons test. **F** Peripheral blood C3 levels in patients with ICH stratified by hemorrhage location and ventricular involvement. Lobe, *n* = 10; basal ganglia without intraventricular hemorrhage (IVH), *n* = 37; basal ganglia with IVH, *n* = 17. Statistical analysis was performed using one-way ANOVA followed by Tukey’s multiple comparisons test. **G** Correlation analysis between circulating C3 levels and hematoma volume. Lobe, *n* = 10; basal ganglia without IVH, *n* = 37; basal ganglia with IVH, *n* = 17. Correlation was analyzed using Pearson’s correlation analysis. *P < 0.05, ** *P* < 0.01, *** *P* < 0.001, **** *P* < 0.0001 and ns, not significant

Given that both differential expression and cell-cell communication analyses indicated upregulation of complement-related pathways in ependymal cells and suggested ependymal cells as a source of complement signaling, we next examined complement activation after ICH at the histological level, molecular level, and in terms of clinical relevance. We firstly analyzed the cellular distribution of *C3* and *C1q* expression across different cell populations in scRNA-seq dataset. The results showed that *C3* was predominantly expressed in ependymal cells (34.7%), whereas *C1qa*, *C1qb*, and *C1qc* were mainly expressed in microglia and showed minimal expression in ependymal cells (Additional file 3). Immunofluorescence analysis showed that C3 protein expression was markedly increased in FOXJ1⁺ ependymal cells in the injured brain. (Fig. 7A, Additional file 4). Western blot analysis of mouse brain tissues demonstrated a significant upregulation of C3 and C1q protein levels after ICH compared with sham controls (Fig. 7B-D). Furthermore, ELISA measurements showed a marked increase in the levels of the membrane attack complex (MAC) in brain tissues after ICH, indicating activation of the complement pathway (Fig. 7E).

To assess the clinical relevance of complement activation, peripheral blood C3 levels were analyzed in patients with ICH. Compared with patients with lobar hemorrhage, those with basal ganglia hemorrhage showed higher serum C3 levels irrespective of the presence of intraventricular hemorrhage, while serum C3 levels did not differ significantly between basal ganglia hemorrhage patients with and without intraventricular involvement. (Fig. 7F). Importantly, peripheral C3 levels showed a positive correlation with hematoma volume in basal ganglia hemorrhage patients (Fig. 7G), suggesting an association between complement activation and hemorrhage severity.

### BST2 regulates the activation of complement pathway in ependymal cells after ICH

**Fig. 8:**
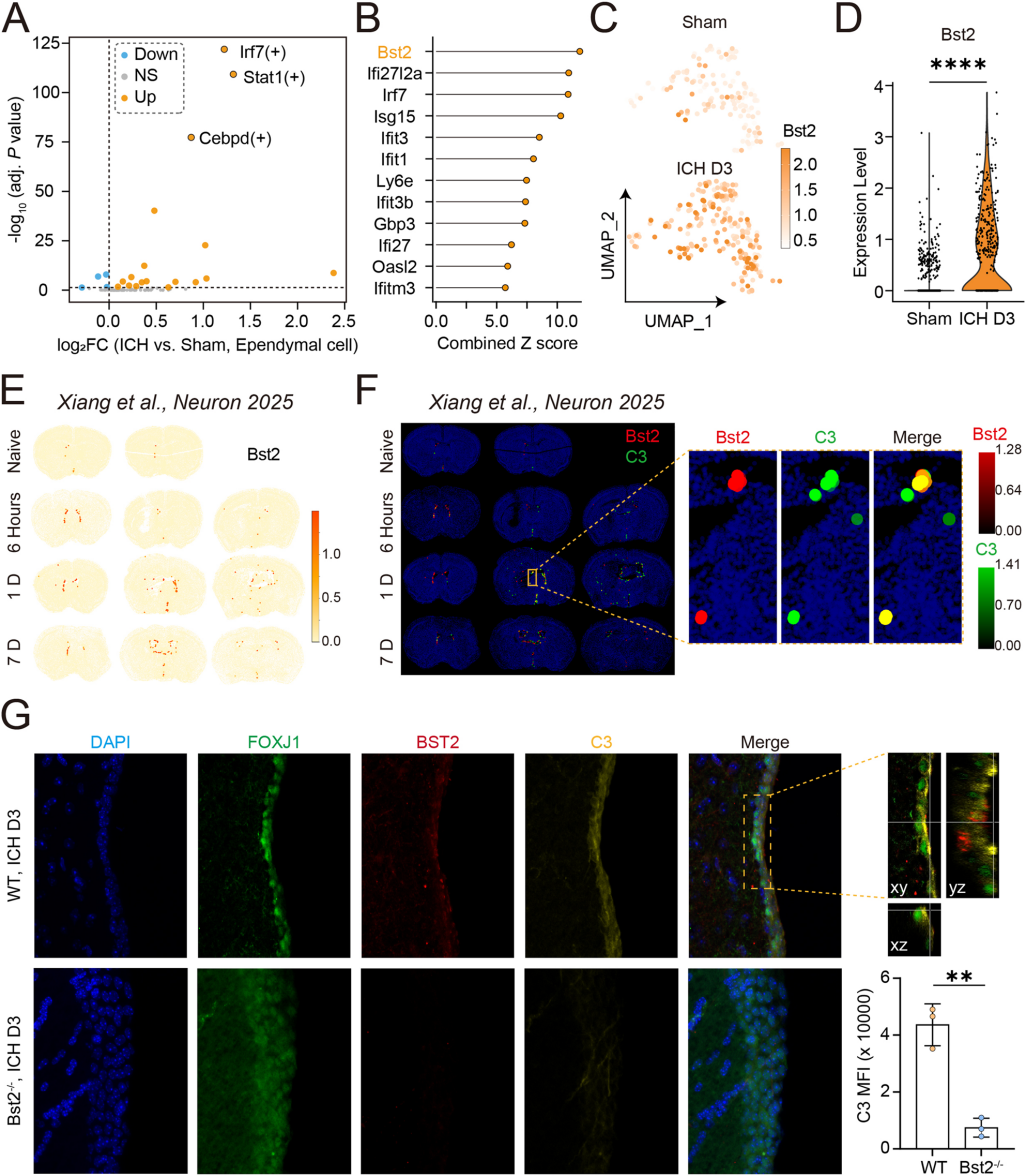
C3 expression regulated by BST2 after ICH. **A** Volcano plot of transcription factor activities in ependymal cells under sham and ICH (3-day) conditions based on single-cell transcriptomic analysis. **B** Combined Z score of IFN related genes based on single-cell transcriptomic analysis. **C**, **D** UMAP plots showing the expression of *Bst2* in ependymal cells from sham and ICH (3 days) groups based on single-cell transcriptomic analysis. Violin plots showing the expression level of *Bst2* in ependymal cells from sham and ICH (3 days) groups based on single-cell transcriptomic analysis. **E** Spatial transcriptomic visualization showing *C3* expression in ependymal cells following ICH at multiple time points. Data were adapted from Xiang et al., *Neuron*, 2025 [14]. **F** Spatial transcriptomic maps showing the expression of *Bst2* (red) and *C3* (green) in brain sections following ICH at multiple time points, with overlapping regions indicating spatial co-expression of *Bst2* and *C3*. Data were adapted from Xiang et al., *Neuron*, 2025 [14]. **G** Representative immunofluorescence images showing DAPI (blue), FOXJ1 (green), BST2 (red), and C3 (yellow) in ependymal cells of WT and *Bst2*^*−/−*^ mice at 3 days after ICH. Orthogonal slice views (xy, xz, yz) from Imaris 3D Slicer are shown to illustrate the spatial localization of the signals. Quantification of C3 mean fluorescence intensity (MFI) in the ependymal region is shown on the right (*n* = 3 per group). Statistical analysis was performed using Student’s t test. * *P* < 0.05, ** *P* < 0.01, *** *P* < 0.001, and **** *P* < 0.0001

To further investigate the mechanisms by which ependymal cells participate in complement activation after ICH, we performed transcription factor analysis based on ependymal cells from single-cell transcriptomic data, which revealed increased activity of the interferon-related transcription factor *Irf7* as well as the JAK-STAT signaling pathway-associated transcription factor *Stat1* (Fig. 8A). A combined Z score, integrating both log2 fold changes and the proportion of expressing cells, identified *Bst2* as the most prominently altered gene associated with complement pathway activation. (Fig. 8B-D). Consistently, spatial transcriptomic analysis demonstrated increased *Bst2* expression in ependymal cells, similar to *C3* expression (Fig. 5E), peaked at day 1 after ICH and declined by day 7 (Fig. 8E). Further spatial co-expression analysis indicated co-expression of *Bst2* and *C3*, with higher *C3* expression observed in *Bst2*⁺ cells (Fig. 8F). Based on these observations, using *Bst2*-deficient mice, we observed a reduction in C3 expression, with additional immunofluorescence analyses supporting its association with ependymal cells (Fig. 8G). Interestingly, in *Bst2*^*−/−*^ mice, we observed fewer IBA1^+^ cells surrounding the peri-ependymal region, suggesting decreased microglia accumulation in this area (Additional file 5E, F). Correspondingly, although *Bst2*^*−/−*^ mice did not exhibit improved cognitive performance in the Y-maze test, ventricular asymmetry was significantly alleviated compared with WT ICH mice (Additional file 5G, H). Taken together, these results suggest that BST2 regulates the activation of C3 complement pathway in ependymal cells following ICH.

## Discussion

In this study, by integrating single-cell and spatial transcriptomic analyses, we demonstrated that ICH induces functional alterations in ependymal cells, including reduced ciliary motility and enhanced inflammation responses; furthermore, we identified BST2-C3 axis through which ependymal cells may regulate activation of the complement pathway.

Neuroinflammation is a major driver of secondary brain injury after ICH, and traditional studies have primarily focused on the perihematomal parenchyma, with emphasis on resident microglia and infiltrating peripheral immune cells [31]. More recent studies have increasingly shifted attention to the borders of CNS, including ventricular system, highlighting that these regions are not merely passive barriers but active hubs of neuro-immune interactions [32, 33]. As an important component of ventricular system, ependymal cells play essential roles and have been implicated in immune-related processes in multiple disease models, including Alzheimer’s disease and aging [29, 30]. Consistent with these observations, our data show that ependymal cells acquire immune and inflammatory features after ICH, including transcriptional programs associated with immune cell chemotaxis and immune activation. In addition, we observed increased accumulation of immune cells, including infiltrating populations, in the ependymal-adjacent region after ICH. Spatial transcriptomic analysis further suggested that these cells are mainly of peripheral myeloid origin, although their precise identity will require further experimental validation. Together, these findings extend the current understanding of post-ICH neuroinflammation by highlighting ependymal cells as important and previously underappreciated participants in inflammatory responses at CNS boundaries following hemorrhagic injury.

Through cell-cell communication analysis, we found that ependymal cells emit multiple signaling factors that were received by immune cells including microglia, macrophages, and neutrophils, with complement component C3 being particularly prominent. Extensive clinical and experimental evidence has demonstrated that complement components such as C1q and C3 are rapidly induced after hemorrhage and contribute to leukocyte recruitment, microglial activation, and secondary tissue damage [34]. Consistent with these observations, we found an activation of complement pathway in ependymal cells, especially C3, suggesting that complement pathway may represent an important mechanism through which ependymal cells participate in post-hemorrhagic inflammatory activation. Previous studies have largely considered astrocytes as the primary source of C3 [35–37], yet recent evidence indicates that under neuroinflammatory conditions, ependymal cells can acquire astrocyte-like reactive features, such as high GFAP expression [38]. These findings raise the possibility that ependymal cells may also serve as a source of C3. While the relative contributions of ependymal cells and other cells including astrocytes to complement activation after ICH remain to be fully established, our data suggest that ependymal cells may also contribute to C3 production in this context. By contrast, for C1q, our single-cell data indicate that its expression is predominantly enriched in immune cells, although whether ependymal cells can also serve as a source of C1q warrants further investigation. In addition, although our clinical analysis identified an association between plasma C3 levels and basal ganglia hemorrhage, circulating C3 levels cannot be considered equivalent to C3 secretion from ependymal cells. Therefore, further studies are needed to more fully define the role of ependymal cells in complement pathway activation after ICH.

To elucidate the mechanisms underlying C3 upregulation in ependymal cells, we performed transcription factor analysis and identified significantly increased activity of *Irf7* and *Stat1*, both of which are central regulators of innate immune responses. IRF7 is a key mediator of type I interferon (IFN) signaling, whereas STAT1 is a core component of the JAK-STAT pathway that orchestrates innate immune activation [39–41]. A recent whole-brain cell atlas of aging mouse revealed high expression of interferon-related genes in ependymal cells [30]. Among interferon-related genes, bone marrow stromal cell antigen 2 (BST2) exhibited the most pronounced change. BST2 (also known as tetherin) is an IFN-inducible protein [42]. Previous studies have established a clear association between BST2 and NF-κB signaling [42]. Notably, recent work has shown that boundary astrocytes can regulate microglial responses after ischemic stroke through a BST2/NF-κB/C3 axis [43]. Using *Bst2*-deficient mice, we also observed a marked reduction of C3 expression in ependymal cells following *Bst2* deletion. Interestingly, *Bst2* deficiency was associated with reduced lateral ventricular asymmetry after ICH, whereas Y-maze performance remained comparable to that in wild-type mice. This inconsistency may in part due to the fact that BST2 is not restricted to ependymal cells but is also expressed in other cell types, including astrocytes, as suggested by recent work identifying BST2-high astrocytes in the injured CNS [43]. Given that we used a global *Bst2* knockout model, the extent to which these phenotypes are specifically attributable to ependymal cells remains uncertain. Furthermore, our inference is based primarily on immunofluorescence co-localization and should therefore be interpreted cautiously. Whether the reduction in C3 signal within ependymal cells reflects a direct, cell-autonomous effect of *Bst2* deletion or arises secondarily from broader changes in surrounding cell populations or the inflammatory microenvironment remains unclear. In addition, the impact of Bst2 deletion on ependymal cell biology itself has not yet been defined. Future studies using ependymal cell-specific manipulation of BST2 will be needed to clarify its direct role and to more rigorously evaluate downstream consequences, including ventricular asymmetry, cognitive outcomes, and immune cell infiltration. Collectively, these findings support the notion that BST2 functions as an upstream regulator of complement pathway activation in ependymal cells. However, definitive elucidation of these mechanisms will require future studies employing cell type-specific genetic manipulation and comprehensive mechanistic analyses.

In line with previous reports describing the transcriptional heterogeneity of ependymal cells [44], our analysis likewise identified EPEN subclusters enriched for cilia-related and developmental programs; however, because our data were generated in the setting of ICH, these subclusters may reflect injury-associated states in addition to baseline heterogeneity, and notably, they retained their distinct transcriptomic identities while showing a shared inflammatory response after injury. In parallel, we observed significant downregulation of genes and pathways related to ciliary motility and neurodevelopment in ependymal cells after ICH, indicating compromised structural and functional integrity of the ventricular lining. Ependymal cilia are essential for cerebrospinal fluid circulation and maintenance of ventricular homeostasis, and their dysfunction has been closely linked to ventricular asymmetry and hydrocephalus [45], a condition in which cognitive decline is a prominent clinical manifestation. Accordingly, we assessed cognitive function using the Y-maze test, and observed marked ventricular asymmetry at later stages after ICH, which was accompanied by cognitive impairment. Although, our data do not directly establish a causal relationship between reduced ciliary motility and cognitive impairment, these findings suggest that ependymal dysfunction may be associated with delayed cognitive decline following hemorrhagic injury. However, this relationship should be interpreted cautiously, as impaired Y-maze performance may also result from neuronal injury and its sequelae rather than from ciliary dysfunction alone. In light of previous studies linking altered ciliary function to changes in ventricular volume [46], and ventricular enlargement to cognitive decline [47], our findings are more consistent with an indirect model in which ciliary dysfunction may disrupt cerebrospinal fluid circulation and ventricular homeostasis, contribute to ventricular enlargement, and thereby secondarily relate to cognitive decline. Notably, recent studies have linked IFN signaling to ventricular asymmetry and ependymal dysfunction [48, 49], raising the possibility that the inflammation-associated alterations in ependymal cells identified in this study may be mechanistically related to impaired ciliary motility. Nevertheless, further validation of key cilia-related components and more direct functional studies will be required to determine whether ciliary dysmotility contributes causally to post-ICH ventricular pathology and cognitive outcomes.

## Conclusion

**Fig. 9:**
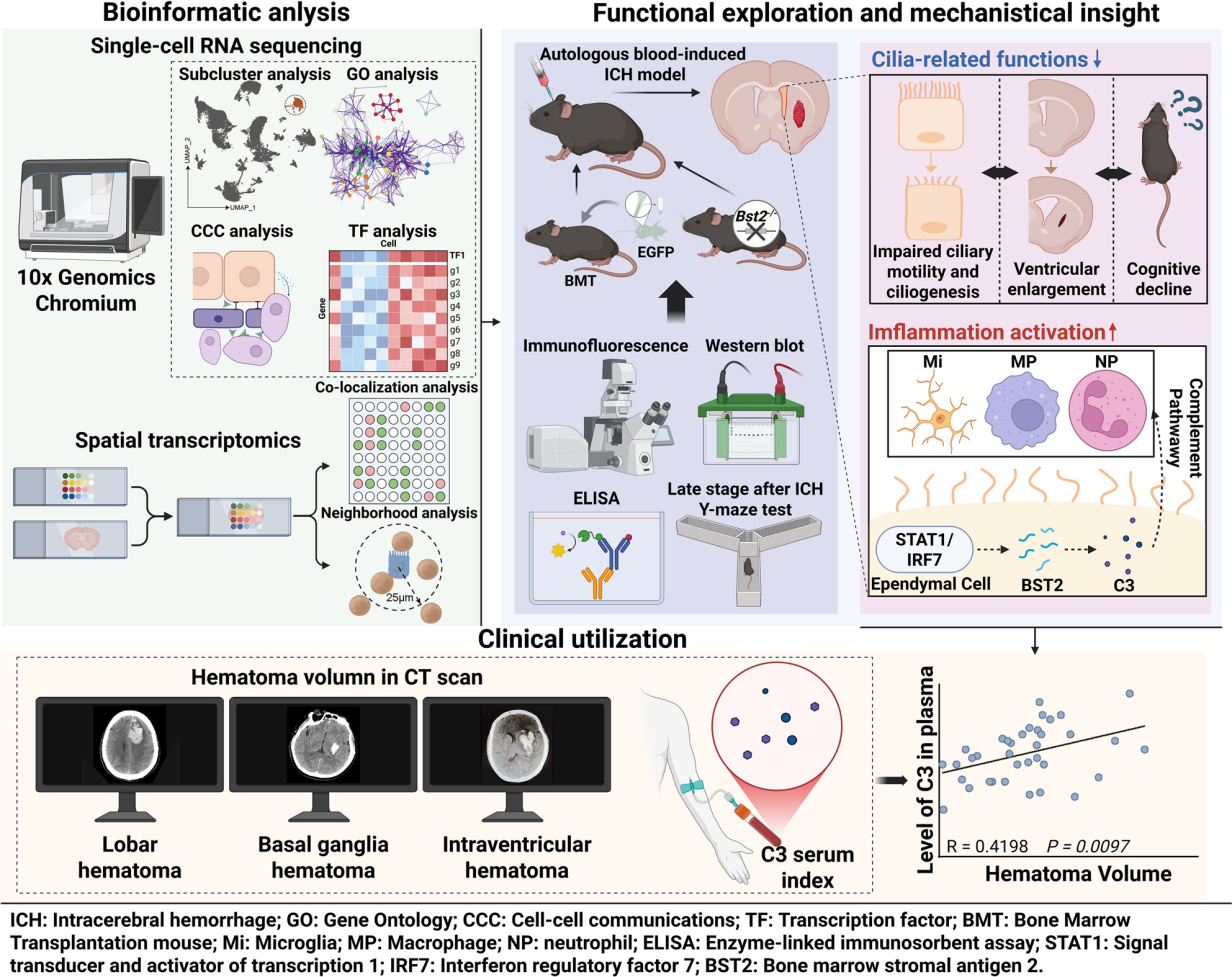
Schematic diagram of inflammatory activation of ependymal cells after intracerebral hemorrhage

In summary, our study identifies ependymal cells as active participants in neuroinflammation following ICH and highlights a potential BST2-C3 axis underlying complement-related signaling. Furthermore, we demonstrate an association between impaired ependymal ciliary motility and ventricular asymmetry, as well as a link between ventricular asymmetry and cognitive dysfunction (Fig. 9). These findings expand the current understanding of ICH pathology beyond the brain parenchyma and suggest that targeting ependymal immune signaling may represent a promising therapeutic strategy to mitigate secondary brain injury after hemorrhagic stroke.

## Supplementary Information

Additional file 1. Baseline characteristics of patients with intracerebral hemorrhage analyzed in this study.

Additional file 2. (A) Spatial transcriptomic maps showing the expression of representative ependymal cell markers, including *Ccdc153*, *Foxj1*, and *Cd24*. Data were adapted from Xiang et al., *Neuron*, 2025 [14]. (B) Spatial transcriptomic maps showing the expression of disease-associated microglia (DAM) -associated genes in ependymal cells, including *B2m*, *H2-D1*, *Cd52*, *Cd63*, *Cd68*, *Cd9*, *Ctsb*, *Ctsd*, *Lyz2*, *Spp1*, *Tyrobp*, and *Trem2*. Data were adapted from Xiang et al., *Neuron*, 2025 [14]. (C) Representative immunofluorescence images showing FOXJ1 (green) and CTSB (red) expression and quantification of CTSB mean fluorescence intensity (MFI) in the ependymal-adjacent region.

Additional file 3. Donut charts showing the proportions of *C1qa*-, *C1qb*-, *C1qc*-, and *C3*-expressing cells among the indicated brain cell populations in ICH group.

Additional file 4. Representative immunofluorescence images demonstrating increased C3 expression in ependymal cells after ICH and quantification of C3 mean fluorescence intensity (MFI) in the ependymal-adjacent region.

Additional file 5. (A) Schematic illustration of Bst2 knockout strategy and PCR-based genotyping validation. (B) Spatial transcriptomic mapping of microglia and ependymal cells in mouse brain sections at the indicated time points. Data were adapted from Xiang et al., *Neuron*, 2025 [14]. (C) Quantification of the number of nearby microglia in the ependymal-adjacent region on the ipsilateral and contralateral sides at the indicated time points after ICH. (D) Relative proportions of immune-related cell populations in the ipsilateral ependymal-adjacent region at 1 day after ICH. (E-F) Representative immunofluorescence images showing FOXJ1 (green) and IBA1 (red) in the ventricular region of WT sham mice, WT mice at 3 days after ICH, and *Bst2*^*-/-*^ mice at 3 days after ICH, and quantification of IBA1-positive cells adjacent to the ventricular region in these groups (*n* = 4/group). LV, lateral ventricle. Statistical analysis was performed using one-way ANOVA followed by Tukey’s multiple comparisons test. (G-H) Quantification of the degree of lateral ventricular asymmetry and Y-maze performance shown as the percentage of time spent in the novel arm in sham, WT (14 days after ICH), and *Bst2*^*-/-*^ (14 days after ICH) groups. Sham, *n* = 8; WT, *n* = 11; *Bst2*^*-/-*^, *n* = 10. Statistical analysis was performed using one-way ANOVA followed by Tukey’s multiple comparisons test.

## Data Availability

All raw data used in this manuscript are available on reasonable request.

## Abbreviations

ICH: Intracerebral hemorrhage
CNS: Central nervous system
IVH: Intraventricular hemorrhage
SPF: Specific pathogen-free
GOBP: Gene Ontology Biological Process
WT: Wild type
ELISA: Enzyme-linked immunosorbent assay
MAC: Membrane attack complex
ANOVA: One-way analysis of variance
ASC: Astrocyte
CPC: Chronic plexus cells
EC: Endothelial cells
EPEN: Ependymal cells
FB: Fibroblasts
Mi: Microglia
MP: Monocyte/macrophages
NB: Neuroblast
NEUR: Neurons
NP: Neutrophils
OEG: Olfactory ensheathing glial cells
OL: Oligodendrocyte
PC: Pericytes
T: T cells
VLMC: Vascular and leptomeningeal cells
VSMC: Vascular smooth muscle cells
DAM: Disease-associated microglia
IFN: Interferon
BST2: Bone marrow stromal cell antigen 2
GPI: Glycosylphosphatidylinositol
LV: Lateral ventricle
