# Supplementary figures and images for: Ependymal cell inflammatory activation in response to intracerebral hemorrhage
Source: J Neuroinflammation. 2026 Apr 14;23:162. doi: 10.1186/s12974-026-03809-z (PMC13200338; doi:10.1186/s12974-026-03809-z)

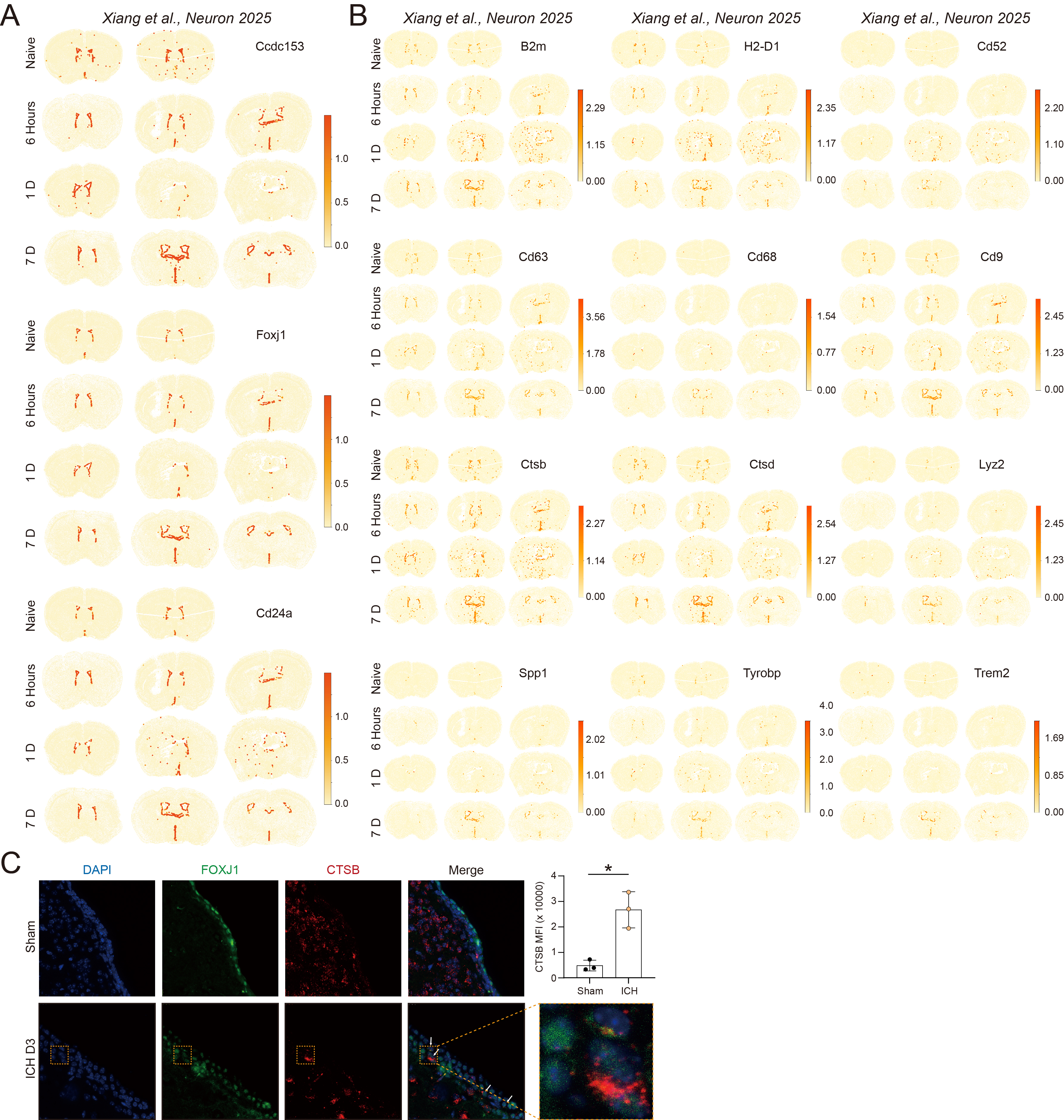

Supplement: Supplementary file 2 — Additional file 2. (A) Spatial transcriptomic maps showing the expression of representative ependymal cell markers, including Ccdc153, Foxj1, and Cd24. Data were adapted from Xiang et al., Neuron, 2025 [14]. (B) Spatial transcriptomic maps showing the expression of disease-associated microglia (DAM) -associated genes in ependymal cells, including B2m, H2-D1, Cd52, Cd63, Cd68, Cd9, Ctsb, Ctsd, Lyz2, Spp1, Tyrobp, and Trem2. Data were adapted from Xiang et al., Neuron, 2025 [14]. (C) Representative immunofluorescence images showing FOXJ1 (green) and CTSB (red) expression and quantification of CTSB mean fluorescence intensity (MFI) in the ependymal-adjacent region. [file 12974_2026_3809_MOESM2_ESM.jpg]

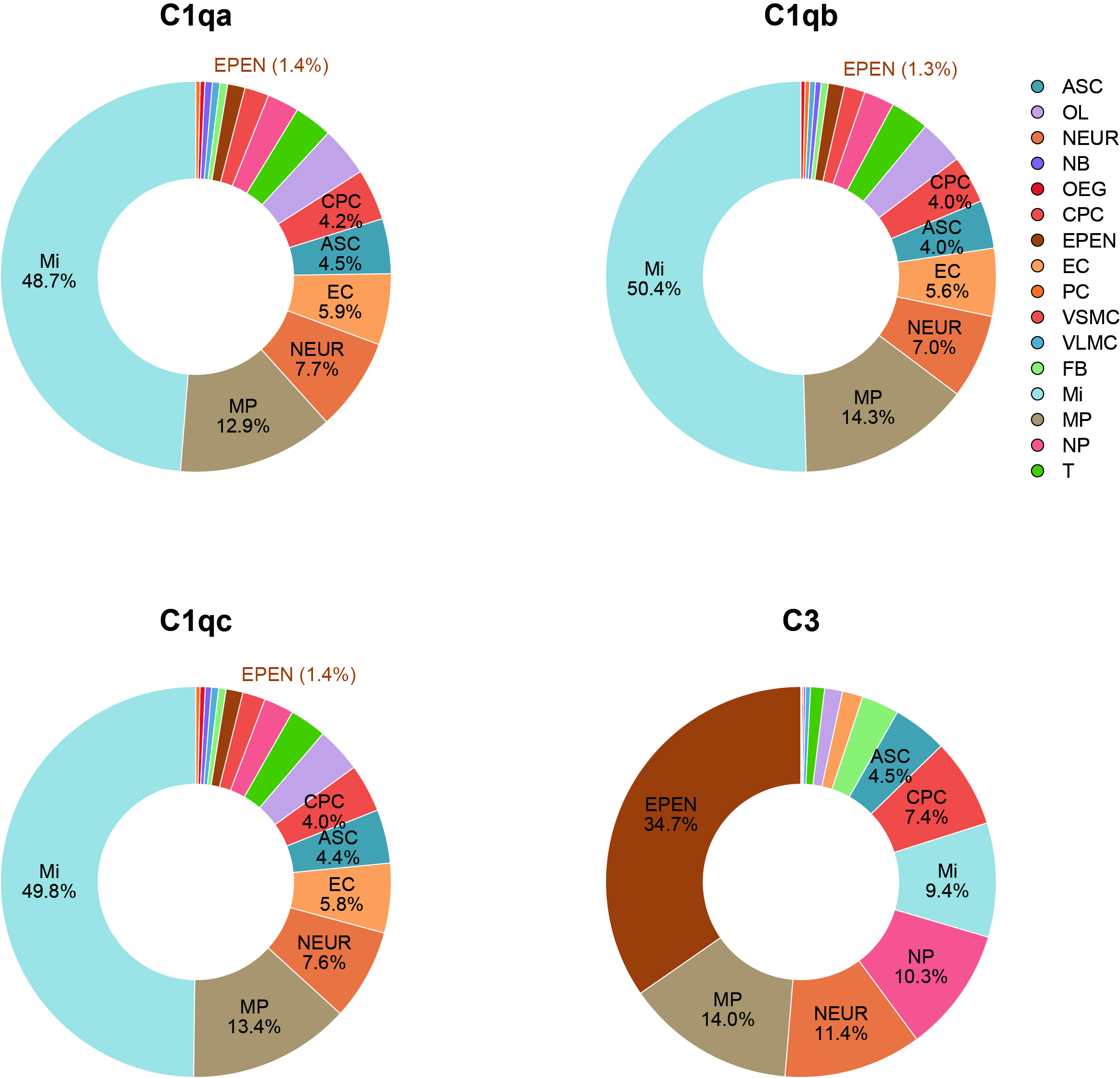

Supplement: Supplementary file 3 — Additional file 3. Donut charts showing the proportions of C1qa-, C1qb-, C1qc-, and C3-expressing cells among the indicated brain cell populations in ICH group. [file 12974_2026_3809_MOESM3_ESM.jpg]

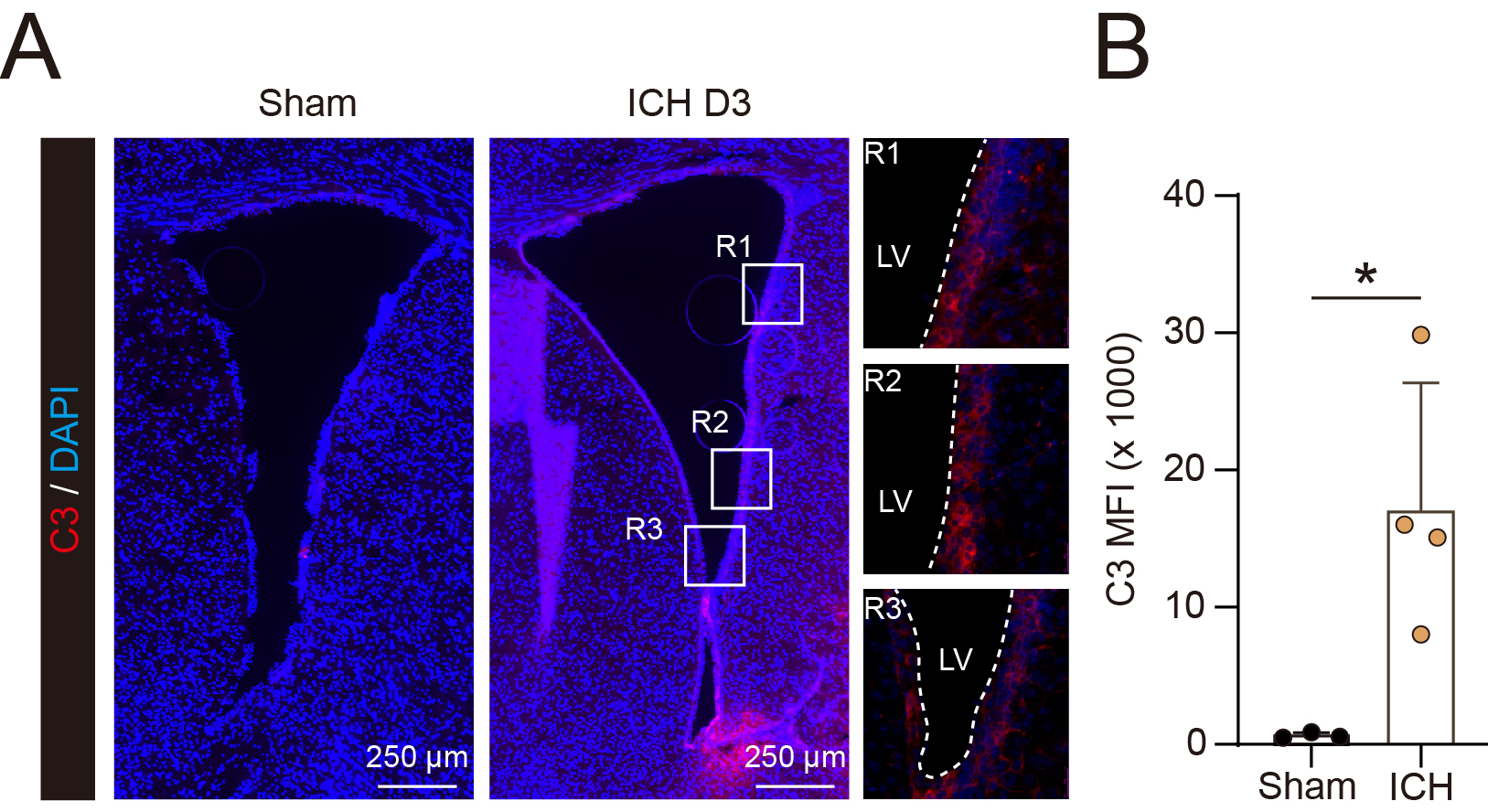

Supplement: Supplementary file 4 — Additional file 4. Representative immunofluorescence images demonstrating increased C3 expression in ependymal cells after ICH and quantification of C3 mean fluorescence intensity (MFI) in the ependymal-adjacent region. [file 12974_2026_3809_MOESM4_ESM.jpg]

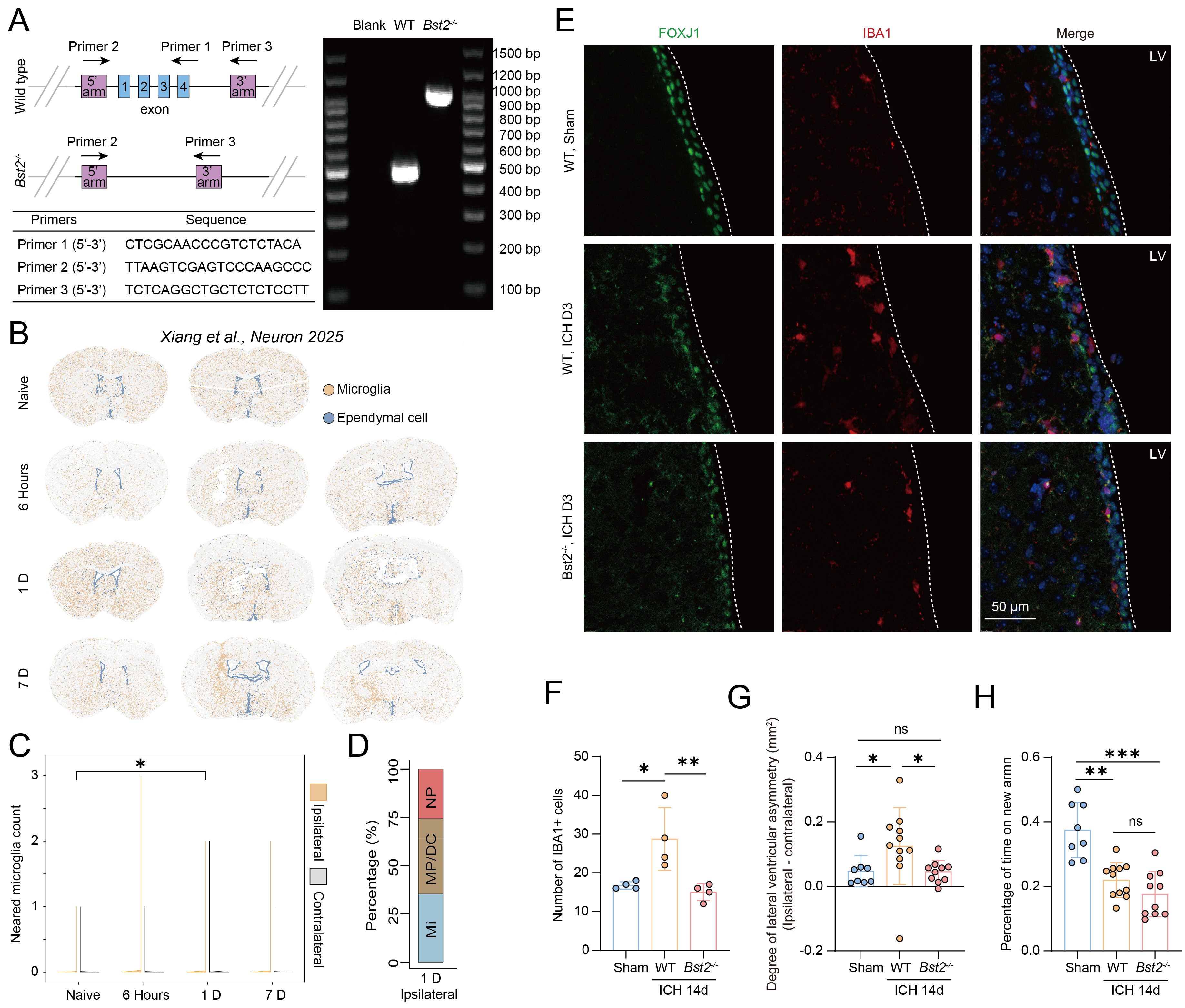

Supplement: Supplementary file 5 — Additional file 5. (A) Schematic illustration of Bst2 knockout strategy and PCR-based genotyping validation. (B) Spatial transcriptomic mapping of microglia and ependymal cells in mouse brain sections at the indicated time points. Data were adapted from Xiang et al., Neuron, 2025 [14]. (C) Quantification of the number of nearby microglia in the ependymal-adjacent region on the ipsilateral and contralateral sides at the indicated time points after ICH. (D) Relative proportions of immune-related cell populations in the ipsilateral ependymal-adjacent region at 1 day after ICH. (E-F) Representative immunofluorescence images showing FOXJ1 (green) and IBA1 (red) in the ventricular region of WT sham mice, WT mice at 3 days after ICH, and Bst2-/- mice at 3 days after ICH, and quantification of IBA1-positive cells adjacent to the ventricular region in these groups (n = 4/group). LV, lateral ventricle. Statistical analysis was performed using one-way ANOVA followed by Tukey’s multiple comparisons test. (G-H) Quantification of the degree of lateral ventricular asymmetry and Y-maze performance shown as the percentage of time spent in the novel arm in sham, WT (14 days after ICH), and Bst2-/- (14 days after ICH) groups. Sham, n = 8; WT, n = 11; Bst2-/-, n = 10. Statistical analysis was performed using one-way ANOVA followed by Tukey’s multiple comparisons test. [file 12974_2026_3809_MOESM5_ESM.jpg]
